# Epidemiology of recurrent pulmonary tuberculosis by bacteriological features of 100 million residents in China

**DOI:** 10.1186/s12879-022-07622-w

**Published:** 2022-07-22

**Authors:** Hui Jiang, Jinfeng Yin, Fangchao Liu, Yuxia Yao, Chao Cai, Jiying Xu, Lijun Zheng, Chendi Zhu, Junnan Jia, Xu Gao, Wangli Xu, Weimin Li, Guolong Zhang

**Affiliations:** 1 Beijing Chest Hospital, Capital Medical University, Beijing, 101149 China; 2 Beijing Tuberculosis and Thoracic Tumor Research Institute, Beijing, 101149 China; 3 Institute of Tuberculosis Control and Prevention, Henan Center for Disease Control and Prevention, Henan, 450016 China; 4 Beijing Youan Hospital, Capital Medical University, Beijing, 100069 China; 5 Beijing Municipal Key Laboratory of Clinical Epidemiology, School of Public Health, Capital Medical University, Beijing, 100069 China; 6 Beijing Key Laboratory in Drug Resistance Tuberculosis Research, National Tuberculosis Clinical Lab of China, Beijing Tuberculosis and Thoracic Tumour Research Institute, Beijing, 101149 China; 7 School of Public Health, Peking University, Beijing, 100191 China; 8 School of Statistics, Renmin University of China, Beijing, 100872 China

**Keywords:** Pulmonary tuberculosis, Recurrence, Risk factors, Clinical diagnosed

## Abstract

**Background:**

Recurrence continues to place significant burden on patients and tuberculosis programmes worldwide, and previous studies have rarely provided analysis in negative recurrence cases. We characterized the epidemiological features of recurrent pulmonary tuberculosis (PTB) patients, estimated its probability associated with different bacteriology results and risk factors.

**Methods:**

Using 2005–2018 provincial surveillance data from Henan, China, where the permanent population approximately were 100 million, we described the epidemiological and bacteriological features of recurrent PTB. The Kaplan–Meier method and Cox proportional hazard models, respectively, were used to estimate probability of recurrent PTB and risk factors.

**Results:**

A total of 7143 (1.5%) PTB patients had recurrence, and of 21.1% were bacteriological positive on both laboratory tests (positive–positive), and of 34.9% were negative–negative. Compared with bacteriological negative recurrent PTB at first episodes, the bacteriological positive cases were more male (81.70% vs 72.79%; P < 0.001), higher mortality risk (1.78% vs 0.92%; P = 0.003), lower proportion of cured or completed treatment (82.81% vs 84.97%; P = 0.022), and longer time from onset to end-of-treatment. The probability of recurrence was higher in bacteriological positive cases than those in bacteriological negative cases (0.5% vs 0.4% at 20 months; P < 0.05).

**Conclusions:**

Based on patient’s epidemiological characteristics and bacteriological type, it was necessary to actively enact measures to control their recurrent.

**Supplementary Information:**

The online version contains supplementary material available at 10.1186/s12879-022-07622-w.

## Background

Recurrence of tuberculosis (TB) is defined as patients who have previously been treated for TB, and were declared cured or treatment completed at the end of their most recent course of treatment, and now are diagnosed with a recurrent episode of TB (either a endogenous reactivation of a previous infection or a new episode of TB caused by reinfection) [1]. According to WHO global estimates, approximately 430,000 previously treated patients experienced bacteriologically confirmed or clinically diagnosed recurrence in 2015, representing 7% of all notified TB cases [2]. In detail, approximately 5% of patients with drug-susceptible tuberculosis have a recurrence after 6 months of first-line therapy, and approximately 20% of patients have a recurrence after 4 months of short-course therapy, even when regimens include a fluoroquinolone [3]. Therefore, recurrence has become the core stumbling block to achieve WHO’s target of eradicating TB in 2035.

In China, extensive studies have been published on TB recurrence; however, most of these studies have focused on pathogens of bacteriological positive cases caused by exogenous reinfection or endogenous relapse as well as the relationship between recurrence and drug resistance [4–6]. However, these studies have rarely provided analysis in negative recurrence cases, which consists of 35.4% of the total recurrence of TB in a previous study [7] and unlike the bacteriological positive cases, most of them are lack of timely diagnosis and treatment. Therefore, it is worthwhile to conduct a comprehensive study on this topic to elucidate the factors that may be associated with the different features of bacteriological positive and negative recurrence cases.

Henan, with approximately 100 million residents, is one of the most populous provinces in China [8], and the number of new cases is about 60,000 each year and accounts for almost 10% of the national TB patients of China mainland [9]. In this retrospective study, we describe the epidemiological features of all recurrent pulmonary tuberculosis (PTB) cases from 2005 to 2008 in Henan province, estimate the recurrence probability of bacteriological positive and negative cases, and explore the risk factors of recurrent PTB.

## Methods

### Data sources

The information of PTB cases from January 1, 2005 to December 31, 2018, which were reported to the Tuberculosis Information Management System (TBIMS) as the national TB surveillance system within 24 h after diagnosis [10], were collected and included basic demographics, time of illness onset and diagnosis, laboratory outcomes, supervisor mode, and treatment outcomes.

### Case definitions

Based on WHO guideline, clinical-diagnosed PTB patients [1] and bacteriologically-confirmed patients were defined respectively. We defined clinical-diagnosed PTB as bacteriological negative PTB, clinical diagnosis was based on TB-specialized chest imaging, supplemented by epidemiological investigation, clinical manifestation (coughing, expectoration ≥ 2 weeks, or hemoptysis), or results of an immunology test (tuberculin skin test and/or interferon gamma release assay), and effective experimental treatment using antituberculosis drugs for 2 months. Bacteriological positive PTB was based on laboratory evidence (sputum smear, culture and Genxpert) of infection with *Mycobacterium tuberculosis* (*M.*TB). Both were defined as PTB patients in our study.

We identified patients with at least two episodes of PTB reported in the TBIMS by matching records using any of the following screening criteria: (1) having highly similar patient names; (2) having highly similar patient names and home addresses; (3) having highly similar patient names, home addresses, and birth dates. We considered patients to have recurrent TB if they experienced at least two independent episodes of TB. An independent episode was defined as a case wherein a patient who was previously diagnosed with PTB and received anti-tuberculosis treatment was declared cured at the end of the course or completion of treatment but was subsequently diagnosed with PTB again. Additionally, patients with at least two independent episodes of recurrent laboratory-confirmed PTB were classified into this group. We also excluded the following PTB patients: (1) not cured; (2) treatment was not completed at the end of their most recent course of treatment.

Because of the retrospective study design, there was no active follow-up for all of the cases. We defined the passive follow-up time as the time after the end of the initial tuberculosis treatment until an event (recurrence) or the censored date (December 31, 2018). To aid comparison with other published studies, we also looked at the proportion of recurrence among a subset of all pulmonary cases, bacteriologically confirmed pulmonary cases who completed treatment.

### Data analysis

Descriptive analyses of recurrent PTB cases were performed using a descriptive method to analyze continuous variables and categorical variables. The Kaplan–Meier method was used to estimate the recurrence curve of PTB. The probability of first and second recurrences of PTB were both calculated. Subgroups of interest were compared using the log rank test. The risk factors associated were explored using Cox proportional hazard models. Only the first episode of recurrence was included in the Cox models. Unadjusted hazard ratios (HRs) and adjusted HRs (aHRs) were estimated from a univariable analysis and multivariable analysis, respectively.

All statistical analyses were performed with R (version 3.3.0). All statistical tests were two-sided with a significance level of *p* < 0.05.

## Results

**Fig. 1:**
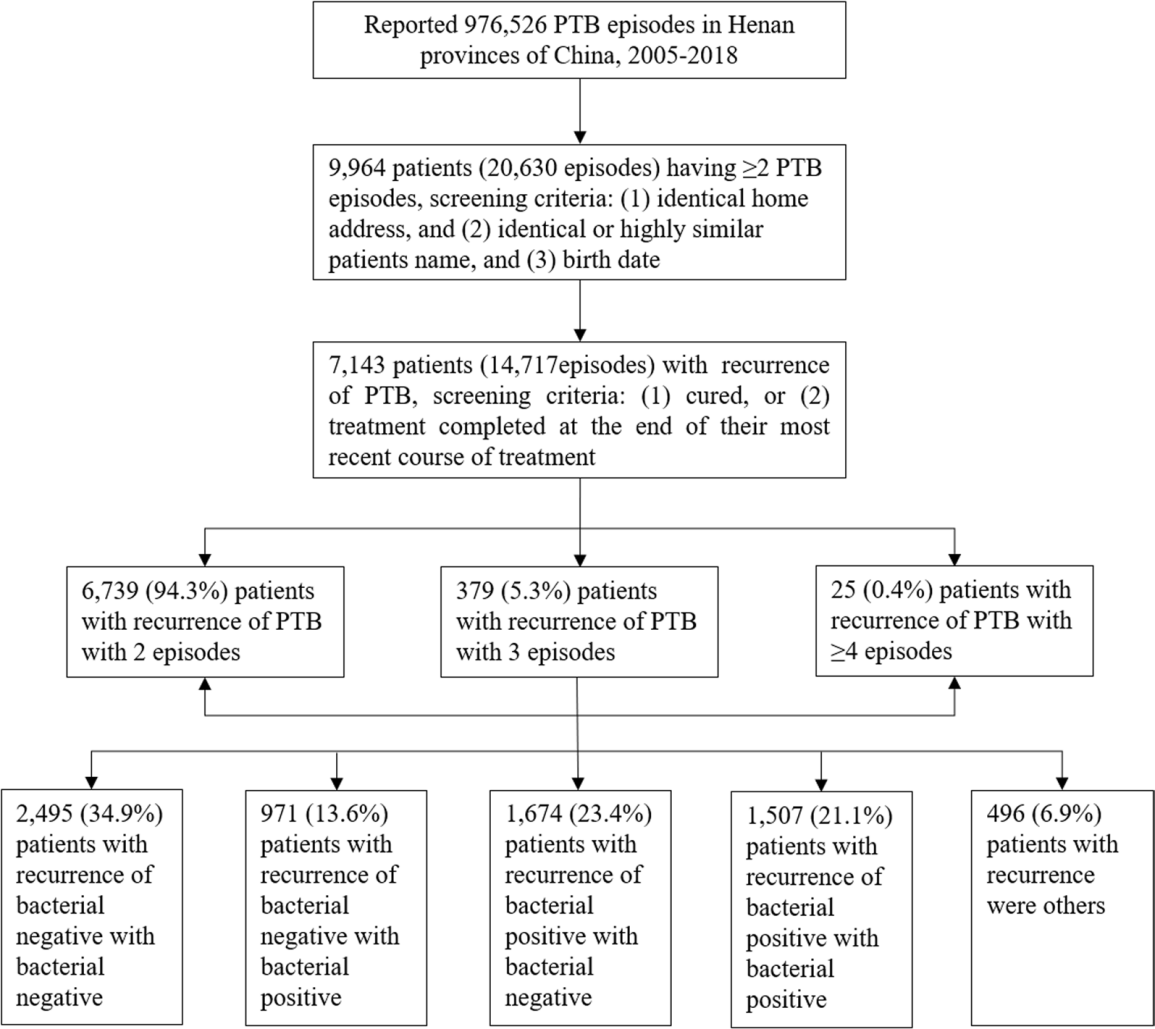
Flowchart showing screening and analysis of patients with recurrent PTB from the national TB surveillance database in Henan province, China from 2005 to 2018

From 2005 to 2018, a total of 976,526 PTB episodes occurring in Henan province were reported to the TBIMS. Excluding single TB episodes, extrapulmonary tuberculosis, and non-independent episodes, 7143 patients (having 14,717 [1.5%] episodes) were identified as having recurrent PTB. Among all PTB and bacteriological confirmed PTB cases, who completed treatment for the first episode, the proportion of recurrence was 1.8% and 2.2% respectively. Among the 7143 recurrent patients, 94.3% (6739) of these patients had two episodes and 5.7% had more than two episodes: 379 (5.3%) patients had three episodes and 25 (0.4%) had at least four episodes. Bacteriological results revealed that 21.1% of patients with recurrence tested bacteriological positive on both laboratory tests, 34.9% tested bacteriological negative on both tests, 23.4% tested bacteriological positive on the first test and bacteriological negative on the second, and 13.6% tested bacteriological negative on the first test and bacteriological positive on the second (Fig. 1; Additional file 1: Fig. S1).

### Demographic characteristics

The overall male to female ratio was 3.4:1. The median age at primary onset of all patients with recurrent PTB was 54 years (interquartile range [IQR]: 40–64), which was higher than that of non-recurrent cases (48 [IQR: 28–63]). In addition, the median ages of patients with one recurrence, two recurrences, and three or more recurrences were 57 (IQR: 42–67), 59 (IQR: 48–68), and 63 (IQR: 54–68), respectively. 50.7% of recurrent cases received enhanced supervision, 49.2% received a directly observed treatment short course chemotherapy (DOTS), and 0.1% performed self-medication. Additionally, the proportion of death in recurrent cases was higher than that in non-recurrent cases, and the proportions of death with one recurrence, two recurrences and three recurrences were 1.4%, 1.9%, and 4.0%, respectively (Table 1). Compared with bacteriological negative recurrent PTB at first episodes, bacteriological positive cases had higher proportion of male patients (81.70% vs 72.79%; *p* < 0.001) and DOTS management mode (99.08% vs 5.45%; *p* < 0.001). And higher levels of mortality risk (1.78% vs 0.92%; *p* = 0.003), lower proportion of cured or treatment completed patients (82.81% vs 84.97%; *p* = 0.022), and longer time from onset to course end were also observed for bacteriological positive recurrent PTB at first episodes (Table 2).

**Table 1:** Characteristics of pulmonary tuberculosis (PTB) patients with different recurrence frequency, Henan province, 2005–2018

Characteristic	No recurrence (n = 961,809)	Recurrence PTB			
		Primary onset (n = 7143)	Recurrence 1 (n = 6739)	Recurrence 2 (n = 379)	Recurrence ≥ 3 (n = 25)
Sex					
Male	676,648 (70.4)	5505 (77.1)	5505 (77.2)	313 (77.8)	18 (72.0)
Female	285,161 (29.6)	1638 (22.9)	1638 (22.8)	91 (22.2)	9 (28.0)
Median (IQR)	48 (28–63)	54 (40–64)	57 (42–67)	59 (48–68)	63 (54–68)
Age group (years)					
0–14	9407 (1.0)	22 (0.3)	5 (0.1)	0 (0.0)	0 (0.0)
15–29	246,098 (25.6)	1198 (16.8)	993 (14.7)	35 (9.2)	3 (12.0)
30–44	180,552 (18.8)	1065 (14.9)	912 (13.5)	39 (10.3)	0 (0.0)
45–59	218,838 (22.8)	2150 (30.1)	1819 (27.0)	118 (31.1)	7 (28.0)
≥ 60	306,504 (31.9)	2708 (37.9)	3010 (44.7)	187 (49.3)	15 (60.0)
Unknown	410 (0.0)	0 (0.0)	0 (0.0)	0 (0.0)	0 (0.0)
Median of time delay (days, range)					
Time from onset to first hospital visit	13 (2–30)	11 (1–31)	11 (1–30)	12 (1–31)	2 (0–16)
Time from onset to confirm	23 (10–40)	14 (30–59)	27 (14–44)	31 (16–50)	28 (17–36)
Time from onset to course end	208 (192–234)	215 (198–249)	223 (200–274)	240 (203–284)	220 (207–255)
Time from hospitalization to course end	185 (182–198)	189 (184–215)	199 (183–249)	210 (184–258)	211 (191–246)
Management mode					
Enhanced supervision	479,607 (49.9)	3624 (50.7)	3733 (55.4)	198 (52.2)	11 (44.0)
DOTS	450,977 (46.9)	3511 (49.2)	2922 (43.4)	175 (46.2)	13 (52.0)
Self-medication	8119 (0.8)	7 (0.1)	12 (0.2)	3 (0.8)	1 (4.0)
Unknown	23,106 (2.4)	1 (0.0)	72 (1.1)	3 (0.8)	0 (0.0)
Outcome					
Cured	292,466 (30.4)	3212 (45.0)	1749 (26.0)	85 (22.4)	5 (20.0)
Treatment completed	483,794 (50.3)	3931 (55.0)	3783 (56.1)	208 (54.9)	15 (60.0)
Death	8112 (0.8)	0 (0.0)	92 (1.4)	7 (1.9)	1 (4.0)
Unknown	177,437 (18.4)	0 (0.0)	1115 (16.6)	79 (20.8)	4 (16.0)

Data are presented as n (%) of patients unless otherwise indicated

*DOTS* directly observed treatment short course chemotherapy, *IQR* interquartile range

**Table 2:** Characteristics of recurrent PTB patients at first episodes

Characteristic	Recurrent PTB at first episodes		P value
	Bacteriological negative cases (n = 3639, 52.44%)	Bacteriological positive cases (n = 3300, 47.56%)	
Sex			< 0.001
Male	2649 (72.79)	2696 (81.70)	
Female	990 (27.21)	604 (18.30)	
Age group			0.233
0–14 years	18 (0.49)	2 (0.06)	
15–29 years	636 (17.48)	506 (15.33)	
30–44 years	520 (14.29)	499 (15.12)	
45–59 years	1055 (28.99)	1054 (31.94)	
≥ 60 years	1410 (38.75)	1239 (37.55)	
Treatment history			0.577
New case	3463 (95.16)	3147 (95.36)	
Retreatment case	176 (4.84)	154 (4.67)	
Time from the completion to recurrent tuberculosis (month, range)	17.6 (8.3–32.5)	17.5 (8.3–35.5)	0.121
Median of time delay (days, range)			
Time from onset to first hospital visit	10 (1–29)	13 (1–34)	< 0.001
Time from onset to confirm	26 (13–48)	32 (15–62)	< 0.001
Time from onset to course end	213 (197–245)	216 (199–252)	< 0.001
Time from hospitalization to course end	189 (184–213)	189 (184–215)	0.592
Management mode			< 0.001
Enhanced supervision	3397 (94.41)	29 (0.89)	
DOTS	196 (5.45)	3234 (99.08)	
Self-medication	5 (0.14)	1 (0.03)	
Outcome^a^			< 0.001
Cured	749 (20.82)	1134 (34.74)	
Treatment completed	2308 (64.15)	1569 (48.07)	0.022
Death	33 (0.92)	58 (1.78)	0.003
Others	549 (15.26)	539 (16.51)	

^a^Treatment outcome at first recurrent episodes for recurrent PTB patients

### Probability of recurrence

**Fig. 2:**
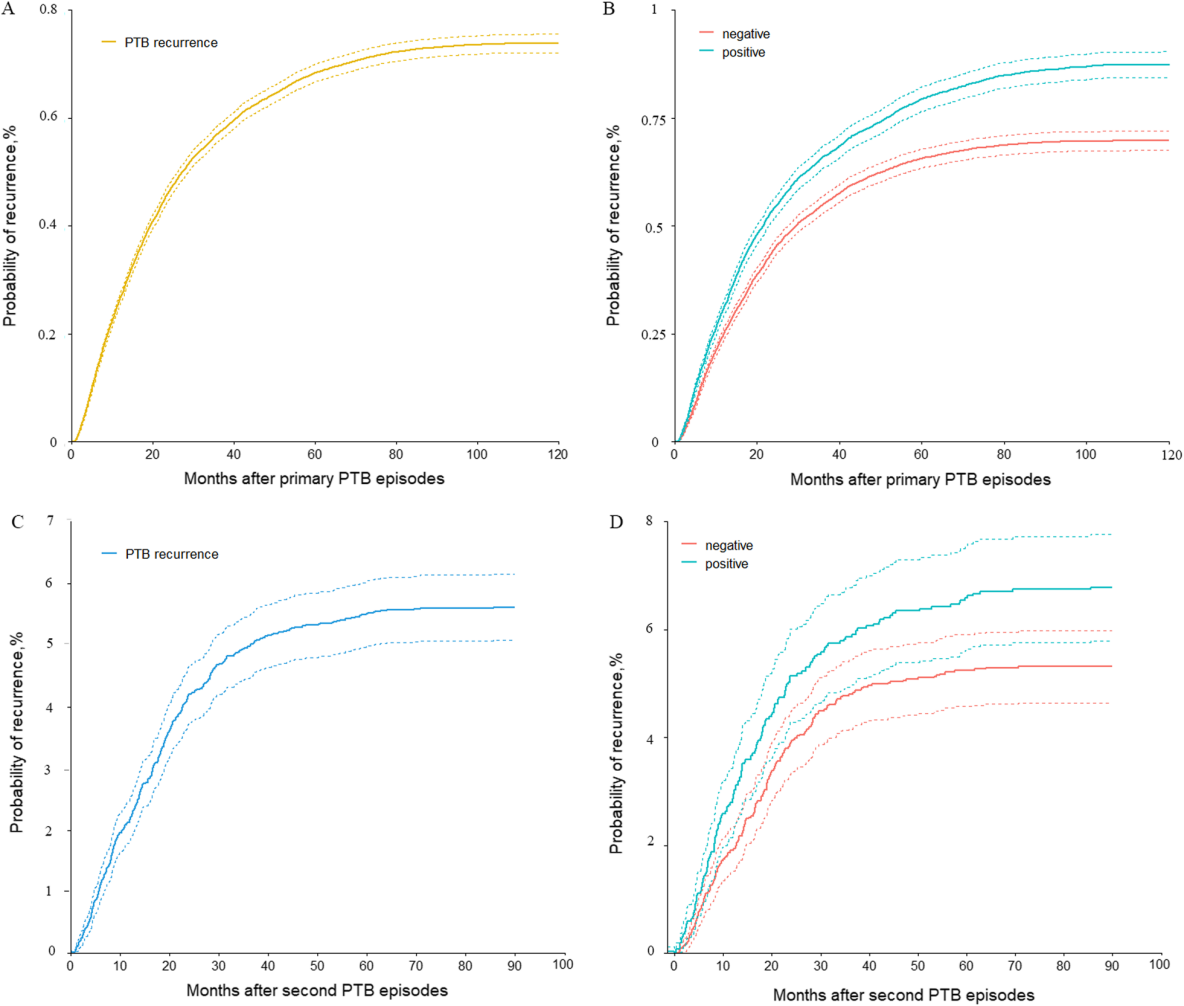
The probability of PTB recurrence after primary PTB diagnosis in Henan province, China from 2005 to 2018. **A** Probability of recurrence in primary PTB patients. **B** Probability of recurrence in primary PTB patients with bacteriological positive and negative cases. **C** Probability of recurrence after first recurrence. **D** Probability of recurrence after first recurrence with bacteriological positive and negative cases

The median times from the completion of first episode to recurrent tuberculosis were 17.6 months (IQR: 8.3–33.9), 17.6 months (IQR: 8.3–32.5), and 17.5 months (IQR: 8.3–35.5) for all pulmonary cases, bacteriological negative cases, and bacteriological positive cases, respectively (*p* = 0.121). Figure 2 depicted the probabilities of the recurrences of the three types of TB cases. Specifically, after the primary episode of PTB (Fig. 2A, B), the recurrences of all PTB and bacteriological positive PTB were similar along with the probabilities of 0.4%, 0.6%, and 0.7% at 20, 40, and 60 months, respectively, and then remained stable after 60 months. The recurrence probabilities of bacteriological negative cases were slightly lower than the positive cases (*p* < 0.05), but still showed an increasing trend.

Compared to the first scenario, during the median time of 15.7 (IQR: 7.5–24.4) months after the second PTB episode in patients with recurrence, the recurrence probabilities were much higher regardless of the types of PTB cases (Fig. 2C, D). All PTB and bacteriological positive cases shared similar probabilities of 3.6%, 5.2%, and 5.6% at 20, 40, and 60 months, respectively. And bacteriological negative PTB had significantly lower probabilities at the same time point. After 62 months, all probabilities kept stable like that in the first scenario.

### Risk factors associated with recurrence

Compared to female, males had higher risk of recurrence with aHRs of 1.29 (95% CI 1.22–1.36). Patients with older age had higher risk of recurrence, compared to the 0–14 years group (reference group), the aHR was 2.32 (95% CI 1.49–3.62) for patients aged 45–59 years, and was 2.07 (95% CI 1.32–3.22) for subjects aged ≥ 60 years (*p* < 0.001). Bacteriological positive cases also exhibited consistently higher risk of recurrence, and the aHRs was 1.27 (95% CI 1.21–1.33) comparing to the negative cases. A longer time from illness onset to cure was associated with greater risk of recurrence, compared to the group of cases with time from illness onset to cure < 6 months (reference group), the aHR was 7.01 (95% CI 5.43–9.04) for cases with time from illness onset to cure between 6 and 12 months, and was 8.32 (95% CI 6.34–10.93) for cases with time from illness onset to cure > 12 months (*p* < 0·001) (Table 3).

**Table 3:** Hazard ratios (HRs) by characteristics of recurrent PTB cases, by univariate and multivariable analysis, Henan province, 2005–2018

	Number of all cases	Number of Recurrent cases	Univariate		Multivariable^a^	
			HRs (95% CI)	*P* value	Adjusted HRs (95% CI)	*P* value
Sex						
Female	286,799	1638	1.00		1.00	
Male	682,153	5505	1.42 (1.34–1.49)	< 0.001	1.29 (1.22–1.36)	< 0.001
Age group, year						
0–14	9429	22	1.00		1.00	
15–29	247,296	1198	2.08 (1.36–3.17)	< 0.001	1.18 (0.76–1.85)	0.449
30–44	181,617	1065	2.52 (1.65–3.84)	< 0.001	1.43 (0.92–2.24)	0.109
45–59	220,988	2150	4.19 (2.75–6.38)	< 0.001	2.32 (1.49–3.62)	< 0.001
≥ 60	309,212	2708	3.77 (2.48–5.74)	< 0.001	2.07 (1.32–3.22)	0.001
Farmer						
No	180,805	1077	1.00		1.00	
Yes	788,139	6058	1.27 (1.19–1.36)	< 0.001	0.98 (0.90–1.06)	0.631
Residence						
Urban	178,955	1248	1.00		1.00	
Rural	780,090	5843	1.07 (1.00–1.14)	0.025	0.99 (0.92–1.07)	0.855
Bacterial test results						
Negative	522,340	3639	1.00		1.00	
Positive	382,335	3300	1.25 (1.19–1.31)	< 0.001	1.27 (1.21–1.33)	< 0.001
Time from illness onset to first hospital visit (month)						
0–1	640,371	5263	1.00		1.00	
1–2	118,170	951	0.98 (0.91–1.05)	0.054	0.95 (0.88–1.01)	0.110
2–3	40,780	388	1.16 (1.05–1.29)	0.005	1.09 (0.98–1.22)	0.089
> 3	53,070	534	1.23 (1.12–1.35)	< 0.001	1.04 (0.94–1.16)	0.426
Time from illness onset to cure (month)						
0–6	46,647	64	1.00		1.00	
6–12	709,329	6459	7.02 (5.49–8.98)	< 0.001	7.01 (5.43–9.04)	< 0.001
> 12	60,517	611	7.52 (5.81–9.73)	< 0.001	8.32 (6.34–10.93)	< 0.001

*HRs* hazards ratios

^a^The multivariable model adjusted for sex, age, occupation and all the other variables in the table

We then assessed the multivariate analysis of hazard ratios of characteristics of bacteriological positive cases and bacteriological negative cases, respectively. In the multivariate analysis, bacteriological negative patients aged 45–59 years and ≥ 60 years were at high risk for recurrence (aHR: 1.92 [1.19–3.07]; aHR: 1.92 [1.20–3.08]) but bacteriological positive patients only had a higher risk on those aged 45–59 years (aHR: 5.09 [1.27–20.44]). There were no other significant differences in risk factors between the two groups (Table 4).

**Table 4:** Hazard ratios (HRs) by characteristics of recurrent PTB cases stratified by Bacteriology results, by univariate and multivariable analysis

	Bacteriological negative cases				Bacteriological positive cases			
	Univariate HRs (95% CI)	*P* value	Multivariable^a^ Adjusted HRs (95% CI)	*P* value	Univariate HRs (95% CI)	*P* value	Multivariable^a^ Adjusted HRs (95% CI)	*P* value
Sex								
Female	1.00		1.00		1.00		1.00	
Male	1.26 (1.17–1.35)	< 0.001	1.20 (1.12–1.29)	< 0.001	1.53 (1.40–1.67)	< 0.001	1.43 (1.31–1.57)	< 0.001
Age group, years								
0–14	1.00		1.00		1.00		1.00	
15–29	1.21 (0.75–1.93)	0.433	1.01 (0.63–1.63)	0.954	3.11 (0.78–12.47)	0.109	2.55 (0.63–10.23)	0.187
30–44	1.37 (0.85–2.19)	0.190	1.18 (0.73–1.90)	0.492	3.83 (0.96–15.36)	0.058	3.18 (0.79–12.77)	0.103
45–59	2.29 (1.44–3.65)	< 0.001	1.92 (1.19–3.07)	0.007	6.30 (1.57–25.22)	0.009	5.09 (1.27–20.44)	0.022
≥ 60	2.27 (1.42–3.61)	< 0.001	1.92 (1.20–3.08)	0.006	4.88 (1.22–19.52)	0.025	4.01 (0.99–16.07)	0.053
Farmer								
No	1.00		1.00		1.00		1.00	
Yes	1.33 (1.21–1.45)	< 0.001	1.01 (0.91–1.13)	0.833	1.09 (0.98–1.19)	0.102	0.95 (0.85–1.66)	0.381
Residence								
Urban	1.00		1.00		1.00		1.00	
Rural	1.16 (1.06–1.26)	< 0.001	1.08 (0.98–1.20)	0.108	0.93 (0.85–1.01)	0.095	0.90 (0.81–1.00)	0.051
Time from illness onset to first hospital visit (month)								
0–1	1.00		1.00		1.00		1.00	
1–2	0.93 (0.84–1.03)	0.154	0.91 (0.82–1.01)	0.062	0.98 (0.89–1.08)	0.645	0.98 (0.89–1.08)	0.662
2–3	1.13 (0.97–1.32)	0.124	1.08 (0.93–1.27)	0.301	1.12 (0.97–1.29)	0.114	1.10 (0.96–1.27)	0.182
> 3	1.17 (1.02–1.34)	0.029	0.95 (0.81–1.11)	0.524	1.17 (1.04–1.32)	0.009	1.13 (0.98–1.30)	0.082
Time from illness onset to cure (month)								
0–6	1.00		1.00		1.00		1.00	
6–12	5.85 (4.23–8.08)	< 0.001	6.03 (4.32–8.41)	< 0.001	8.88 (5.99–13.17)	< 0.001	8.40 (5.67–12.45)	< 0.001
> 12	8.09 (5.74–11.42)	< 0.001	8.31 (5.79–11.90)	< 0.001	9.85 (6.54–14.83)	< 0.001	8.73 (5.76–13.23)	< 0.001

*HRs* hazards ratios

^a^The multivariable model adjusted for sex, age, occupation and all the other variables in the table

## Discussion

The findings from this study showed the overall proportion of recurrent tuberculosis to be 1.5%. The hazard of recurrent tuberculosis was significantly higher for tuberculosis patients who were male, older age, bacteriological positive cases, and with a longer time from illness onset to cure. Notably, bacteriological positive recurrence cases had higher mortality risk (1.78% vs 0.92%; *p* = 0.003) and lower cure or treatment completement rate (82.81% vs 84.97%; *p* = 0.022) than bacteriological negative cases.

A WHO report estimated that 6.8% of TB cases recurred worldwide in 2019 [11], and recurrence occurs not only in high TB incidence countries, but also in low countries [12–14]. A retrospective study of surveillance data and clinical records in Finland showed 0.6% of TB cases were recurrent from 1995 to 2013 [15], and 1.3% of TB cases were recurrent in Barcelona from 2003 to 2006 [12], and the proportion of recurrent cases between 4.2 and 5.7% in the United States during 1993–2010 [16]. In addition, 5.3% of successfully treated bacteriologically confirmed cases had a recurrence in Shanghai [5], China, and 6.8% in Beijing [6]. In comparison, 2.2% of bacteriological confirmed PTB cases who completed treatment recurred in Henan province from 2005 to 2018. Shanghai and Beijing had more comprehensive PTB case detection, treatment and management strategies, compared with the area of Henan, which helped find more PTB and recurrent patients. For example, the administrative department of Shanghai required all pulmonary tuberculosis patients to test sputum culture, and the pathogenic positive rate to be above 50% [17], and extended free tuberculosis treatment to all migrants [18] (free treatment was not available outside individuals’ originally registered residence in Henan). Of course, differences in enrollment patients, the criteria for defining cases as recurrent TB, and the length of the study varied between studies and limit the comparisons.

In this study, we found male gender and older age were related to recurrence, which were in line with some previous reports [19–22]. We also observed that more proportion of men had bacteriological positive PTB recurrent PTB at first episodes (81.70% vs 72.79%), because men tended to have radiographic abnormalities, positive results on smear microscopy, and culture positivity compared with women [23–25]. The time from illness onset to cure within 6 months was associated with a lower risk of recurrence than that for the period beyond 6 months. The aHR also increased with prolongation of treatment time. This demonstrates that longer treatment periods are associated with a higher chance of recurrence and could be due to the treatment noncompliance [22]. Compared with bacteriological negative PTB cases, bacteriological positive PTB cases were more likely to recur. We found that patients with bacteriological positive results had a longer treatment period and some researches showed that bacteriology-negative PTB can be treated for a shorter duration [26]. During the longer treatment period, because of factors such as adverse reactions, cost and stigma [27], there was a lower treatment completion or cure rate on bacteriological positive patients (82.81% vs 84.97%). Furthermore, incomplete or irregular treatment could increase disease recurrence [22]. The health system needs to pay attention to the case management of recurrent patients to ensure patients finish the treatment or take the drugs irregularly, having a benefit impact on reducing recurrence. In addition, bacteriologically positive PTB presented with more symptoms and were more likely to have cavitary lesions than patients with bacteriologically negative PTB [28]. Cavitation on radiology was the risk factor for tuberculosis recurrence [29], which may make bacteriologically confirmed PTB more likely to recur.

More intriguingly, our study specifically evaluated the recurrence of bacteriological negative cases though it had a lower mortality risk than bacteriological positive recurrence cases as previously reported (0.92% vs. 1.78%) but with a more complicated diagnosis criteria. Even if not bacteriological confirmed, such bacteriology-negative PTB may be hindered in routine clinical practice and with high likelihood of progression to transmissible bacteriology-positive disease if left untreated or treated inappropriately [30]. Recent studies suggested the detection from the perspective of immunology mechanisms including the T cell dysfunction and the transitional changes in interferon (IFN)-γ responses [31], as well as the alterations of MTB-specific IgG profiles [32]. These suggest a potential exploration of immunological biomarkers for the detection of bacteriology-negative PTB cases, which may help increase its detection rate in clinical setting and further reduce the recurrence of PTB.

This study has several limitations. First, although most of the clinically diagnosed PTB were caused by *M.*TB, Non-tuberculosis mycobacteria (NTM) infection cannot be ruled out, because the symptom, image feature and treatment scheme of NTM infection are almost the same as that of *M.*TB infection. Second, we did not obtain *M.*TB and blood samples; therefore, we were unable to distinguish between recurrent cases due to reactivation and those due to re-infection, or to investigate their respective risk factors. Third, we were limited to the information routinely collected in the TBIMS, which at the time did not include potentially important variables such as underlying medical conditions, drug resistance status, and clinical symptoms. Further implementation of clinical and treatment characteristics of recurrent TB stratified by bacteriology results will provide more information to understand recurrence. In addition, due to the lack of patient identification numbers, there may be some misclassification of recurrence cases, which caused an underestimation of the proportion of recurrences. And we do not get the national recurrent data, and there is no literature to report the epidemiological characteristics of recurrent cases in China in the national scale, therefore, we cannot compare it with the national recurrent level.

## Conclusions

In conclusion, our study provides a detailed overview of the epidemiology of PTB recurrence from 100 million residents in China. Our findings suggest although bacteriological positive cases had higher risk of recurrence than bacteriological negative cases, and bacteriological positive recurrence cases had higher mortality risk, it was not neglected the clinical diagnosis PTB recurrence cases. It was necessary for the increase of recurrent cases to actively targeted interventions to control based on its epidemiological characteristics.

## Supplementary Information

**Additional file 1: Figure S1.** Flow chart of showing screening of tuberculosis cases with completed treatment or cured.

## Data Availability

The data comes from the Henan Provincial Center for Disease Control and Prevention. If you want data, you can contact Guolong Zhang. The email is 1296190445@qq.com.
