# Supplementary figures and images for: Epidemiology of recurrent pulmonary tuberculosis by bacteriological features of 100 million residents in China
Source: BMC Infect Dis. 2022 Jul 22;22:638. doi: 10.1186/s12879-022-07622-w (PMC9308209; doi:10.1186/s12879-022-07622-w)

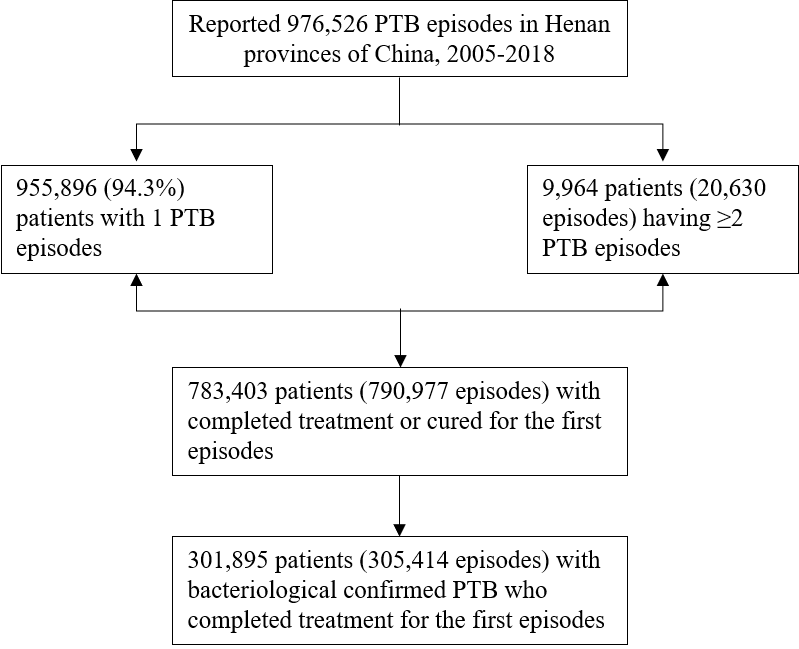


Figure S1: Flow chart of tuberculosis cases with completed treatment or cured.

Supplement: Supplementary file 1 — Additional file 1: Figure S1. Flow chart of showing screening of tuberculosis cases with completed treatment or cured. [file 12879_2022_7622_MOESM1_ESM.docx]
